# Lunasin and Its Epigenetic Impact in Cancer Chemoprevention

**DOI:** 10.3390/ijms24119187

**Published:** 2023-05-24

**Authors:** Agnieszka Kaufman-Szymczyk, Wiktoria Kaczmarek, Krystyna Fabianowska-Majewska, Katarzyna Lubecka-Gajewska

**Affiliations:** 1 Department of Biomedical Chemistry, Faculty of Health Sciences, Medical University of Lodz, 92-215 Lodz, Poland; agnieszka.kaufman-szymczyk@umed.lodz.pl (A.K.-S.); wiktoria.kaczmarek@stud.umed.lodz.pl (W.K.); 2 Faculty of Medicine, Lazarski University, 02-662 Warsaw, Poland; krystyna.fabianowska-majewska@lazarski.pl

**Keywords:** lunasin, chemoprevention, epinutrient, epigenetic anti-cancer therapy, histone acetylation, DNA methylation

## Abstract

Cancer diseases are a leading cause of death worldwide. Therefore, it is pivotal to search for bioactive dietary compounds that can avert tumor development. A diet rich in vegetables, including legumes, provides chemopreventive substances, which have the potential to prevent many diseases, including cancer. Lunasin is a soy-derived peptide whose anti-cancer activity has been studied for over 20 years. The results of the previous research have shown that lunasin inhibits histone acetylation, regulates the cell cycle, suppresses proliferation and induces apoptosis of cancer cells. Thus, lunasin seems to be a promising bioactive anti-cancer agent and a potent epigenetic modulator. The present review discusses studies of the underlying molecular mechanisms and new perspectives on lunasin application in epigenetic prevention and anti-cancer therapy.

## 1. Introduction

In the modern world, cancerous diseases are a leading cause of death worldwide, with many new cases of cancer being diagnosed each year. The World Health Organization (WHO) reports that in 2020, almost 10 million people died due to cancer [1]. It highlights the necessity of searching for new and effective methods of cancer prevention. A healthy body maintains a balance between cell division and cell loss. Any disturbance can result in uncontrolled and excessive proliferation and the development of neoplastic disease. The major cancer risk factors include tobacco use, occupational carcinogens, improper diet, pathogens or environmental carcinogens [2].

To prevent cancer, it is recommended to follow a proper diet, especially one rich in vegetables, roots and legumes. These products contain numerous bioactive compounds with chemopreventive properties. Particularly noteworthy is soy—a legume rich in isoflavones, saponins and peptides with biological activity [3]. The most commonly tested bioactive nutrients in soy with anti-cancer potential are the isoflavones (phytoestrogens) and Bowman–Birk protease inhibitor (BBI) [4,5]. However, exposure to isoflavone mixtures was found to yield various chemopreventive effects, suggesting that other soy components may also play a role in those outcomes [6]. One such component is lunasin, a unique bioactive soy-derived peptide, which appears to be a good candidate as an anti-cancer agent. 

Lunasin was discovered in Japan in 1987 [7], and the first article mentioning lunasin was published in 1999 [8]; it was found that the expression of a lunasin protein in bacterial *Escherichia coli* led to the appearance of aberrant elongated filaments in bacteria and resulted in mitotic arrest and cell death in mammalian cells [8]. Subsequently, lunasin and its biological activities and potential anti-cancer effects have been tested by other several laboratories.

Previous studies have shown that lunasin possesses epigenetic anti-cancer activity, as its anti-mitotic effects have been attributed to its binding to regions of hypoacetylated chromatin [8]. In healthy cells, the level of histone acetylation and deacetylation is balanced. Histone acetylation results in the relaxation of the chromatin structure, which increases gene expression [3,8]. Lunasin has been shown to compete with histone acetyltransferases (HATs) and prevent the attachment of acetyl groups to deacetylated histones of the selected genes, including those with oncogenic potential [3]; thus, lunasin inhibits their overexpression which might reduce the risk of tumor development and progression [9]. Surprisingly, a limited number of HATs inhibitors have been identified and described, knowing that aberrant HAT activities in cell signaling can trigger cancer development. Therefore, lunasin has a chance to become a new agent with epigenetic chemotherapeutic potential.

It is important to remember that epigenetic modifications comprise several components, such as covalent histone modifications (particularly histone acetylation and methylation), DNA methylation and non-coding RNA-related mechanisms. Both histone modifications and DNA methylation should be considered in the context of chromatin structure and control of gene expression. Inactive chromatin contains hypermethylated DNA and histone modifications underlying inactive transcriptional state, such as histone deacetylation. Active chromatin is associated with DNA hypomethylation and active histone marks, including histone acetylation. It has been shown that alterations in histone marks can trigger changes in DNA methylation patterns. On the other hand, DNA methylation results in the recruitment of histone-modifying enzymes that reconfigure chromatin structure [10,11]. The interference between histone modifications and DNA methylation [12], highlighting the dynamic aspect of epigenetic modifications, can have important implications for the epigenetic chemopreventive potential of lunasin. Agents affecting the balance of histone acetylation [13,14,15,16,17,18], including lunasin [9,19], may indirectly drive changes in DNA methylation and subsequently gene expression [9,13,14,15,16,17,18,19]. Lunasin, partly by its epigenetically mediated alterations in gene expression, has been shown to arrest the cell cycle, inhibit cell proliferation and induce apoptosis. 

The present review summarizes and discusses key literature selected from over two decades of research on lunasin. It places particular emphasis on its epigenetic anti-cancer potential, including some new perspectives on lunasin-mediated changes in the expression of genes encoding selected epigenetic enzymes.

## 2. Characterization of Lunasin

### 2.1. Chemical Structure of Lunasin and Its Structure-Related Chemopreventive Activities

**Figure 1:**
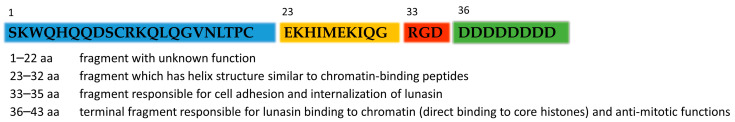
Lunasin structure divided into four fragments and its structure-related activities.

Lunasin is a soy-derived peptide composed of 43 amino acid residues with a molecular weight of 5.5 kDa [20]. It is one of the products of the gene encoding the soy albumin protein (GM2S-1). This gene encodes a signal peptide, a 2S albumin small chain peptide, also known as aspartic acid or lunasin-rich peptide, a linker peptide, a 2S albumin large chain peptide and an 8 kDa methionine-rich protein (8 kDa MRP) [21]. Lunasin peptide comprises four fragments, depicted in Figure 1 [20,22].

The peptide consists of an initial fragment with an unknown role, comprising 22 amino acids (1–22 aa), followed by three more fragments, which appear to be responsible for the specific properties of lunasin. The first, comprising nine amino acids (23–31 aa), most likely has a helical structure and, due to its structural similarity to chromatin-binding proteins, facilitates the attachment of the peptide to core histones. The second fragment is the RGD sequence, comprising three amino acids (32–34 aa, glycine, arginine and glycine), which is responsible for the attachment of lunasin to cancerous cells through extracellular receptors [20] and its internalization in the cell. The RGD cell adhesion motif allows lunasin to reach the nucleus in a few hours [23]. There is also a possibility that lunasin, like other RGD peptides, may induce cell apoptosis through direct activation of caspase-3 [24]. The last fragment is the final sequence consisting of nine aspartic acid residues (35–43 aa), located at the C-terminal end of the peptide; this is responsible for the direct binding of lunasin to chromatin (Figure 1) [20]. 

Studies of the physicochemical and structural properties of lunasin indicate a high degree of disorder in its structure and molecular plasticity, which is strongly dependent on the environment in which it is found. Conformational changes within the molecule are mediated by inter alia the degree of oxidation, electrostatic interactions and the possibility of forming intramolecular disulfide bridges (Cys10-Cys22), and these determine both the stability and biological properties of lunasin [25]. 

The high structural flexibility of lunasin allows the peptide to adopt different structures, able to bind to different partners and make lunasin one of the IDPs (intrinsically disordered peptides). The intrinsic disorder of lunasin is associated with its amino acid composition. Lunasin has a high number of hydrophilic regions in its structure, as well as charged residues, such as its rich aspartic acid end [25]. IDPs, like lunasin, are primarily related to the regulation of transcription, translation and signal transduction and can participate in cellular events, such as DNA condensation, cell cycle, mitosis and apoptosis [26,27].

The aforementioned research indicates that all the parts of lunasin molecule are pivotal to the various mechanisms of its anti-cancer activity. As a consequence of its chemical structure, lunasin shows chemopreventive properties, such as inhibition of histone acetylation, cell cycle arrest, inhibition of cell proliferation and induction of apoptosis [8,23,28,29,30]. 

Cell cycle analysis showed that lunasin causes a G2 cell cycle arrest. Treatment of L1210 leukemia cells with lunasin-enriched flour increased the percentage of cells in the sub-G1 fraction, which is an indicator of DNA fragmentation and loss of cell integrity. A similar effect on internucleosomal DNA fragmentation has been shown in HL60 leukemia cells treated with an RGD peptide. Additionally, the caspase-3 activation in RGD peptide-treated cells was also observed, suggesting that caspase-3 might have a critical role in the execution process of apoptosis induced by RGD [28]. Lunasin-mediated apoptosis is probably a consequence of several events in cells. The expression of proteins associated with the mitochondrial pathway is altered. The expression of the pro-apoptotic Bax is increased by lunasin with a concomitant reduction in anti-apoptotic Bcl-2 expression. Lunasin also increases the expression of the pro-apoptotic form of clusterin, nuclear clusterin (nCLU). As a result of Bax mitochondrial translocation, the release of cytosolic cytochrome c (a main product of mitochondrial permeabilization) is also observed in colon cancer cells [29]. Lunasin also increases the expression and activity of caspases. Initiator caspases-8 and 9 are activated by lunasin, which triggered the activation of caspase-3, an executioner of apoptosis [29,30]. However, in L1210 leukemia cells, also an independent increase in the expression of caspase-3 was observed, in the presence of inhibitors for caspases-8 and 9 [30]. When the RGD cell adhesion motif of lunasin is important to its internalization in the cell and caspases activation, it is probably not required for its binding to the deacetylated NH2-terminal tail of histones [23]. The presence of the polyaspartic residue on the carboxyl end of the lunasin molecule led to its binding to the regions of hypoacetylated chromatin, and it resulted in the mitosis arrest, which might explain the ability of lunasin to induce G2 cell cycle arrest [8]. 

Interestingly, the deletion of the N-terminal, 22-amino acid sequence (with unknown function) of lunasin reduced significantly (by 70%) its ability to bind deacetylated histone H4 in vitro, suggesting that this fragment plays some role in facilitating the binding interactions [23].

### 2.2. Lunasin Abundance in Soybeans and Other Plants

Lunasin is mostly found in soybeans. Its content depends on the soy genotypes, the environmental factors (such as the method of cultivation, temperature, humidity, type of soil and seed maturation) and the type of product in which the soybeans were used [9]. The studies of Joeng and others indicate that the peptide is also present in barley, rye and wheat (Table 1) [20,22,31,32,33]. However, Alaswad and Krishnan’s immunological study performed to confirm the presence or absence of lunasin in the seeds of diverse plants, based on the use of polyclonal antibodies specific to the N-terminal (SKWQHQQDSCRKQLQGVNLT) and C-terminal (CEKHIMEKIQGRGDD) regions of the lunasin peptide, revealed that peptides derived from rye, barley and wheat are not complementary to proteins from soybean [34]. It was shown that the lunasin N-terminal-specific antibody reacted (under less stringent conditions) with a few proteins of different molecular weights present in the seeds of barley, rye and wheat, but none of these cereal proteins reacted (under identical conditions) against the lunasin C-terminal-specific antibody. Moreover, under more stringent conditions, the positive reaction with the lunasin N-terminal antibody was not detected, suggesting the lack of lunasin in these cereals [34]. 

Therefore, further research is needed to clarify the presence or absence of lunasin peptide in other plants than soybean. It seems that according to the previous findings [20,22,31,32,33,34], the proteins from the seeds of cereals should be called ‘lunasin-like peptides’ [34]. 

### 2.3. Digestion and Bioavailability of Lunasin

For a substance to have a therapeutic effect, it requires an appropriate level of bioavailability. Lunasin is protected in the gastrointestinal tract by Bowman–Birk inhibitors as indicated by Cruz-Huerta et al. [35]. Bowman–Birk inhibitors (BBIs) are proteins found mostly in leguminous seeds with a molecular weight of about 8 kDa and a high proportion of disulfide bonds; these are capable of inhibiting chymotrypsin and trypsin at independent binding sites. BBIs originally were classified as anti-nutritional factors because they reduce the digestive efficiency of the main gut intestinal proteases. However, various BBIs have been found to demonstrate chemopreventive and anti-cancer activities. The presence of several disulfide bonds in their conformation makes them extremely stable proteins, resistant to high temperatures (even about 100 °C), a wide pH range (2–12) and to the presence of proteolytic enzymes. As BBIs are naturally occurring protease inhibitors in soy extracts, they might exert a protective effect against lunasin proteolysis by digestive enzymes [36,37].

Mixtures of soybean peptide and its major soybean Bowman–Birk inhibitor (IBB1) in different ratios have been exposed to the action of pepsin and pancreatic enzymes. The best results were obtained with a mixture of lunasin and IBB1 in a 1:2 ratio. The percentage of intact lunasin in the gastric digest was 35.1 ± 2.8 and in the gastric and intestinal digest was 5.3 ± 0.4. Interestingly, the presence of an inactive IBB1 also protects lunasin from complete digestion [35]. 

The bioavailability of lunasin in humans has been investigated by Dia et al. in 2009 [38]. Five healthy men (18- to 25-year old) consumed 50 g of soy protein contained in two meals per day. The total amount of lunasin consumed was 155.5 mg/day. Blood samples were obtained before starting and on the last day of the lasting five-day feeding period. On day 5, blood samples were collected two times: 30 min and 1 h after soy protein consumption. The results indicate that lunasin was not found in any samples collected before the feeding period. However, the mean concentration of lunasin was 66.0 ± 25.4 ng/mL blood after 30 min and 71.0 ± 32.8 ng/mL after 1 h. Assuming that a typical person contains 3 L of plasma, these data show that lunasin was absorbed in 4.5% [38]. The thermostability and good absorption of lunasin are important features that simplify its processing, storage and choosing of potential routes of exposure, including oral administration systems for chemopreventive applications.

## 3. Lunasin and Its Effects on Normal Cells

Traditionally used anti-cancer therapies, such as chemotherapy, radiotherapy or hormonal therapy, are effective in cancer treatment but are significantly harmful to healthy cells. They are exhausting for the patient’s body and cause many undesirable effects. Hence, there is a need to identify new and effective treatment methods. One such candidate is lunasin, a natural bioactive compound with chemopreventive and anti-cancer properties that do not adversely affect healthy cells [23,29,39,40,41,42,43,44,45,46,47].

**Figure 2:**
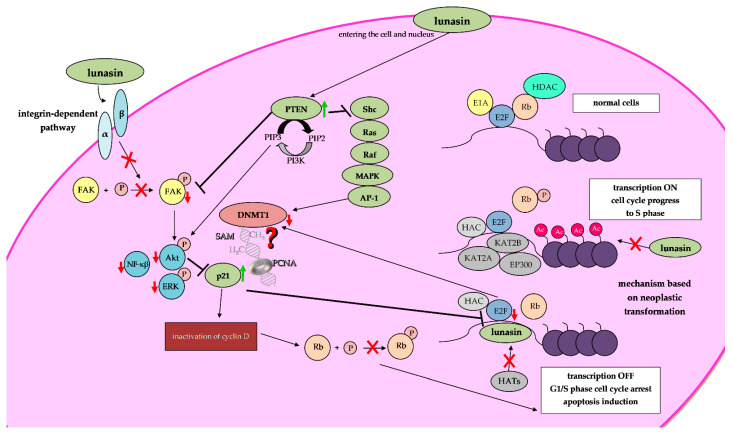
The cell-specific mechanisms of lunasin activity. The proposed potential repressive effects of lunasin, a potent epinutrient, on *DNMT1* transcription and/or DNMT1 activity in cancer cells (middle part of the scheme). Implications of PTEN-mediated negative regulation of intracellular oncogenic signaling pathways, including PI3K/AKT and MAPK/AP-1. PTEN and p21 proteins are negative regulators of AP-1 and E2F, respectively. Those transcription factors (AP-1 and E2F) activate *DNMT1* expression due to the presence of binding sites in the *DNMT1*-regulatory region. Competition of p21 with DNMT1 for the same binding site on PCNA (proliferating cell nuclear antigen). The figure depicts a proposed mechanism of lunasin anti-cancer activity based on the inhibition of integrin signaling and regulation of FAK/AKT/ERK and NF-κB signaling pathways (left part of the scheme) and another mechanism based on an E1A-Rb-HDAC neoplastic transformation model (right part of the scheme). In normal cells in the early G1 phase of the cell cycle (right top part of the scheme), Rb-E2F complex recruits HDACs to maintain the core histones in the repressed/deacetylated state. In the cells being transformed (right middle part of the scheme), during the late G1 phase, E1A (the viral oncogene) inactivates Rb by phosphorylation (Rb-P) and dissociates the Rb-E2F complex, exposing the deacetylated histones to the HATs (i.a., KAT2A, KAT2B, EP300); histone acetylation allows the expression of genes encoding proteins required for S phase and activation of cell cycle progression. In established cancer cell lines (right middle part of the scheme), in which transformation has occurred in the absence of lunasin, HATs acetylated the core histones, turning on cell cycle transcription factors and keeping the acetylated core histones inaccessible and unable to react with added lunasin. Lunasin competes with HATs (i.a., KAT2A, KAT2B, EP300) in binding to hypoacetylated histones (right bottom part of the scheme), repressing transcription that is recognized as abnormal by cells, leading to G1/S phase cell cycle arrest and apoptosis induction. Protein (e.g., Rb) and Protein-P (Rb-P) represent the unphosphorylated and the phosphorylated forms of the protein, respectively. Downregulation (a red down arrow); upregulation (a green up arrow); inhibition (a red X); lunasin as potential modulator of DNA methylation (question mark). Shc, SH2-containing collagen-related proteins; PIP2, phosphatidylinositol (4,5) bisphosphate; PIP3, phosphatidylinositol (3,4,5) trisphosphate; DNMT1, DNA methyltransferase 1; PTEN, phosphatase and tensin homologue; p21 (CDKN1A), cyclin-dependent kinase inhibitor 1A.

Exogenous addition of lunasin to cell cultures, in the absence of carcinogens, does not affect cell morphology and proliferation. However, lunasin was able to act on cells at the stage of division or transformation in the presence of carcinogens. Several studies have proven that lunasin has the capacity for inhibiting cancerous foci formation in cells induced by both chemical carcinogens and viral oncogenes [23,39,47,48,49]. The potential mechanism of lunasin cancer-preventive action was described by the E1A-Rb-histone deacetylase (HDAC) model by Lam et al. in 2003 (Figure 2) [39]. In this study, immortalized mammalian fibroblasts NIH-3T3 were transfected with the viral oncogene E1A. E1A induces cell proliferation by inactivating the tumor-suppressor protein Rb, which controls the G1/S transition by interacting with the E2F promoter and recruiting HDAC to keep the core histones in the deacetylated state. In the presence of lunasin, E1A inactivates Rb through phosphorylation and dissociates the Rb-HDAC complex, exposing the deacetylated core histones in the E2F promoter. Lunasin binds to the deacetylated core histones, competing with HATs, and turns off transcription, perceived as abnormal by the cell, and commits apoptosis. Histone acetylation is turning on E2F cell cycle transcription factors and allows the expression of genes needed for further cell cycle progression. The authors suggest that in established cancer cell lines, in which transformation has occurred in the absence of lunasin, HATs have acetylated the core histones, turning on cell cycle transcription factors and keeping the acetylated core histones inaccessible and unable to react with added lunasin [39].

Studies (Table 2) indicate that both natural (purified from soy products) and synthetic lunasin preparations have no significant negative effect on the growth of non-tumorigenic cells. It has been confirmed that after exogenous application, lunasin internalized into normal cells (NIH-3T3 mouse fibroblast and C3H10T1/2 mouse embryo fibroblasts) [23,39]. Immunostaining experiments showed that lunasin internalizes into the NIH-3T3 cell cytoplasm within 3 h of administration and into the nucleus within 18 h [39]. Whether the lunasin RGD cell adhesion motif is required to internalize into the cells remains to be established. Interestingly, while the RGD motif is required for the internalization of lunasin in C3H10T1/2 mouse embryo fibroblasts, it does not appear to be necessary for internalization in NIH-3T3 cells, suggesting that the internalization mechanism might be cell-specific [23,39].

Recombinant lunasin has also no effects on EA.hy926 cell viability. However, lunasin pretreatment effectively protects endothelial cells against H_2_O_2_-induced cytotoxicity and apoptosis [40].

Overall, some results indicate that lunasin is non-toxic in normal/non-tumorigenic cells, although further studies are needed to determine its safety for chemopreventive applications and therapeutic doses.

**Table 2:** The results of in vitro studies on the lunasin activity in normal cells.

Cell Line	Concentration	Time of Exposition	Type of Lunasin	Proliferation	Ref.
Human normal mammary epithelial MCF-10A	1–320 µM	24–48 h	Synthetic	No significant effect	[41]
Human normal mammary epithelial MCF-10A	5–200 µM	24–72 h	Synthetic	No significant effect	[42]
Normal bronchial epithelial HBE135-E6E7 BEAS-2B	1–100 µM	24–72 h	Natural	No significant effect	[43]
Normal colon fibroblast CCD-33Co	1–100 µM	24 h	Natural	No significant effect	[29,44]
Mouse fibroblast NIH-3T3	10 µM	24–96 h	Synthetic	No significant effect	[39]
Mouse fibroblast NIH-3T3	0.01–10 µM	24–72 h	Synthetic	No significant effect	[45]
Mouse macrophage RAW 264.7	10–50 µM	24 h	Natural	No significant effect	[46]
Mouse macrophage RAW 264.7	0.2–200 μM	24 h	Synthetic	No significant effect	[47]
Permanent endothelial EA.hy926	0.05–120 μM	72 h	Recombinant	No significant effect	[40]

## 4. Chemopreventive Properties of Lunasin

Research on lunasin has been continuing for over 20 years. Various in vitro and in vivo studies indicate that it has high anti-cancer epigenetic potential. Depending on the cell lines and animal models used in studies, different results have been received (Table 3, Table 4 and Table 5). 

### 4.1. Epigenetic Mechanisms of Lunasin Anti-Cancer Activity 

Epigenetics is the field of science focused on the chromatin modifications that impact gene expression, without alterations in the DNA nucleotide sequence. Epigenetic modifications of histones and DNA affect chromatin structure and thus regulate the expression of various genes, including those encoding proteins involved in the control of inter alia the cell cycle, DNA replication, DNA repair and apoptosis [50]. Importantly, epigenetic events are reversible and responsive to different factors and natural or synthetic agents, including dietary compounds. 

Lunasin has been shown to possess potent epigenetic anti-cancer activity. Epigenetic modifications comprise several components, of which histone acetylation is the most studied. In healthy cells, the level of histone acetylation and deacetylation is balanced. There are two groups of enzymes responsible for maintaining the balance between histone acetylation and deacetylation, these being HATs and histone deacetylases (HDACs), respectively. In general, the acetylation of core histones is associated with relaxed chromatin structure and gene transcriptional activity (euchromatin), whereas histone deacetylation with chromatin condensation and repressed transcription (heterochromatin). The studies have shown that in cancerous cells, the aberrant histone code is observed, mediated partly by alterations in HATs and HDACs activity and subsequent changes in histone acetylation and gene expression [10,11]. Therefore, those alterations seem like a good target for epigenetic anti-cancer therapy with natural bioactive compounds. 

**Figure 3:**
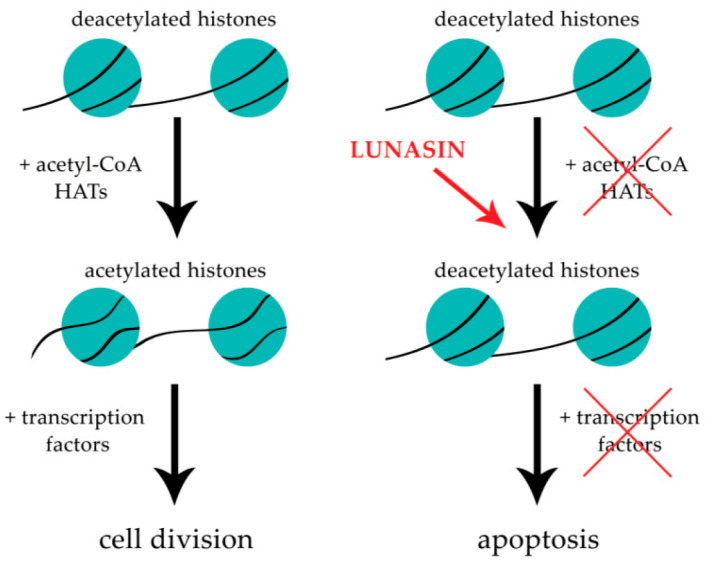
Simplified scheme showing lunasin impact on histone acetylation.

Previous studies indicate that lunasin competes with HATs and prevents the attachment of acetyl groups to deacetylated histones of the selected genes, including those with oncogenic potential (Figure 3). Thus, lunasin inhibits their overexpression which might reduce the risk of tumor development and progression [9]. 

Most importantly, all the epigenetic modifications (i.a., covalent histone modifications, DNA methylation and non-coding RNA-related mechanisms) interfere with each other to create a unique epigenetic network. Therefore, both histone modifications and DNA methylation need to be considered in the context of chromatin structure and control of gene expression. Inactive chromatin contains hypermethylated DNA and histone modifications underlying inactive transcriptional state, i.a., histone deacetylation. On the other hand, active chromatin is associated with DNA hypomethylation and active histone marks, i.a., histone acetylation. The studies suggest that alterations in histone marks can trigger changes in DNA methylation patterns. However, DNA methylation may lead to alterations in histone marks that affect chromatin structure [10,11]. The dynamic interaction between epigenetic modifications, i.a., histone modifications and DNA methylation [12], may influence the epigenetic chemopreventive potential of lunasin. Being a bioactive phytochemical affecting the balance of histone acetylation, lunasin can indirectly cause changes in DNA methylation pattern and thus, gene expression. As such, lunasin has been shown to be responsible for the cell cycle arrest, inhibition of cell proliferation and apoptosis induction, partly by its epigenetically mediated alterations in the expression of genes encoding key proteins controlling cellular processes, specifically those in cancer cells and/or lunasin-sensitive cells. 

Posttranslational modifications of histones such as acetylation, phosphorylation, and methylation usually take place on the ‘tails’ of histones. Strahl and Allis [51] suggest that the acetylation of specific lysine residues in the amino termini of the core histones plays a fundamental role in the regulation of gene transcriptional activity. Thus, the acetylation state of lysine 9 in histone H3 and lysines 5 and 12 in histone H4 has a strong influence on chromatin assembly.

Lunasin has been shown to bind to the deacetylated histones H3 (Lys 9 and 14 sensitive to acetylation) and H4 (Lys 5, 8, 12 and 16 sensitive to acetylation) and inhibit their HAT-mediated acetylation in a dose-dependent manner [32,52]. Interaction of lunasin with core histones, involving inhibition of acetylation on H3-Lys 9 and H4-Lys 8 and 12, may affect the formation of centromere complex and lead to cell cycle arrest in the G1/S phase and apoptosis induction, in ER-independent human breast cancer MDA-MB-231 cells [49,52]. Moreover, the studies revealed that RB upregulation and inhibition of RB phosphorylation may also trigger the observed lunasin-mediated effects on breast cancer cell cycle and cell death [49]. Upon lunasin exposure in MDA-MB-231 cells, downregulation of cyclins D1 and D3, and cyclin-dependent kinases 4 and 6, implicated in cell cycle-related pathways, has been observed as well [52]. This mechanism may also contribute to the G1/S phase cell cycle arrest and inhibition of cancer cell growth [52].

#### 4.1.1. Inhibition of Histone Acetylation upon Lunasin Exposure 

Previous studies suggest that the lunasin fragment EKHIMEKIQG has a helical structure similar to chromatin-binding peptides, allowing lunasin to bind to lysine residues within hypoacetylated histones H3 and H4, thus competing with the following HATs, KAT2A (histone acetyltransferase KAT2A; yGCN5), KAT2B (histone acetyltransferase KAT2B; PCAF) and EP300 (histone acetyltransferase p300; P300) (Figure 2 and Figure 3) [52,53,54]. Alterations in the level of histone acetylation have been observed in lunasin-exposed skin cancer [55,56,57], breast cancer [23,52], prostate cancer [19] and non-small-cell lung cancer (NSCLC) [58] cells.

**Figure 4:**
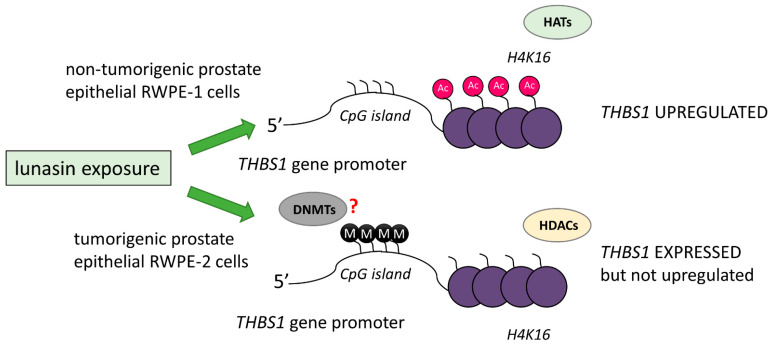
The potential impact of promoter methylation on lunasin-mediated changes in gene expression in non-tumorigenic and tumorigenic cells. Further research is needed to assess lunasin as a potential regulator of DNA methylation processes (indicated by the question mark). RWPE-1, non-tumorigenic prostate epithelial cells; RWPE-2, tumorigenic prostate epithelial cells; *THBS1*, the pro-apoptotic THBS1 (thrombospondin 1) gene; M, hypermethylated CpG island within THBS1 promoter region; Ac, acetylated H4-Lys 16 (H4K16).

Interestingly, upon lunasin exposure, H4-Lys 8 acetylation was found to be suppressed in breast [52] and prostate cancer [19] and in NSCLC (both in lunasin-sensitive H661 and lunasin-insensitive H1299 cells) [58]. The lunasin-mediated acetylation of H4-Lys 12 (by cytoplasmic HAT1) was suppressed both in breast and NSCLC cells. However, lunasin-induced H4-Lys 16 hyperacetylation was noticed in both H661 NSCLC [58] and non-tumorigenic prostate epithelial RWPE-1 [19] cells, but not in tumorigenic prostate epithelial RWPE-2 [19] cells. Moreover, only in lunasin-exposed RWPE-1 cells, increased H4-Lys 16 acetylation was seen within the 5′ end of the pro-apoptotic *THBS1* (thrombospondin 1) gene containing a CpG island; this was found to result in THBS1 upregulation (Figure 4) [19,59]. Galvez et al. suggested that in RWPE-2 cells, the observed histone hypoacetylation and hypermethylation within the mentioned CpG island of the *THBS1* gene could be responsible for the inability of lunasin to increase *THBS1* expression [19]. Galvez et al. suggest that the hypermethylation of the 5′CpG island of the *THBS1* gene in RWPE-2 cells may promote HDAC-mediated H4-Lys 16 deacetylation, leading to chromatin condensation within the *THBS1* promoter and its inaccessibility to basal transcriptional apparatus (Figure 4) [19]. Further studies are needed to confirm the proposed model of epigenetic chemopreventive potential of lunasin (Figure 4). 

Thus, lunasin may prevent H3 and H4 acetylation by HATs and may promote the acetylation of specific lysine residues, triggering changes in gene expression encoding proteins involved in the regulation of cell function processes, including cell cycle and apoptosis.

The control of the cell cycle and cell apoptosis is complex and requires many enzymes (kinases, caspases) and proteins (such as Rb, p21, p27, BAX, Bcl-2, E2F and cyclines), involved in many signaling pathways, such as the integrin signaling pathway (Figure 2) [54,60]. Lunasin has been shown to inhibit integrin signaling and regulate FAK/AKT/ERK and NF-κB signaling networks via binding to integrin [54] (Figure 2). The integrins are ubiquitous receptors that act as a signaling medium between the cytoskeleton and extracellular matrix (ECM) and play a significant role in the regulation of many cellular processes such as gene expression, proliferation, migration and survival. Lunasin cell adhesion motif (RGD) enables its ability to internalize into cells and compete with ECM to interact with integrins. It was found that lunasin inhibits the metastasis of colon cancer cells by direct binding to α5β1 integrin and suppresses FAK/ERK/NF-κB signaling in vitro and in a mouse model. Lunasin downregulates the phosphorylation of FAK and ERK and suppresses constitutive NF-κB activation by increasing the expression of IκB-α (a protein responsible for anchoring NF-κB into the cytoplasm, thereby preventing its translocation into the nucleus) [61]. Lunasin has also been reported to exert anti-inflammatory effects on human macrophages. Lunasin inhibits αVβ3 integrin-mediated proinflammatory markers and downregulates Akt-mediated NF-κB pathways [62].

The lunasin-mediated suppression of the mentioned signaling networks leads to decreased proliferation, apoptosis and reduced metastatic potential in cancerous cells [54] (Figure 2). In different cancer cell models, including leukemia, colon and breast cancer, the epigenetic chemopreventive activity of lunasin, i.e., the modulation of dynamics of histone acetylation–deacetylation, may lead to changes in the expression of the aforementioned genes (Table 3). Lunasin-mediated upregulation of the following genes, *Bax*, *CASP3*, *CASP8*, *CASP9*, *p21*, *p27*, *nCLU*, *MALT1* and *PTEN*, has been observed (Table 3). Concomitantly, upon lunasin exposure, *Bax* downregulation was also observed (Table 3).

#### 4.1.2. Lunasin and Its Potential to Modulate DNA Methylation

Although lunasin has been shown to inhibit histone acetylation, it still remains unclear whether it also affects DNA methylation (Figure 2). If this is the case, this raises the question of whether DNA methylation facilitates histone acetylation or vice versa. Both histone modifications and DNA methylation should be considered in the context of chromatin structure and control of gene expression. The altered histone marks may lead to changes in the activity of DNA-methylating/demethylating enzymes. However, altered DNA methylation patterns can trigger the recruitment of histone-modifying enzymes that modulate chromatin structure [10,11]. Therefore, any interference between histone modifications and DNA methylation [12] should have important consequences for the epigenetic chemopreventive potential of lunasin. Agents affecting the balance of histone acetylation, including lunasin, may indirectly drive changes in DNA methylation and subsequently gene expression. 

Moreover, it has been shown that histone hypoacetylation and hypermethylation observed within the CpG island of the *THBS1* gene can be responsible for the inability of lunasin to affect *THBS1* expression in tumorigenic prostate epithelial RWPE-2 cells [19].

Interestingly, since p21 upregulation has been observed in lunasin-exposed cancerous cells, it is important to mention the potential mechanism that should be considered while assessing lunasin-mediated epigenetic modulation of gene expression by the indirect impact on DNA methylation reaction. Research suggests that many natural bioactive compounds may indirectly trigger the repressive effects on modulation of *DNMT1* transcription and/or DNMT1 activity in cancerous cells, as a result of p21 (CDKN1A) and DNMT1 protein interference [17,63,64,65]. *p21* is a tumor-suppressor gene encoding a protein that competes with DNMT1 for the same binding site on proliferating cell nuclear antigen (PCNA, the homotrimeric ring surrounding DNA) during DNA replication. It disrupts the formation of the DNMT1/PCNA complex, potentially leading to the inhibition of DNA methylation [66,67]. Furthermore, p21 upregulation may result in decreased E2F activity [68], and PTEN re-expression may inhibit AP-1 activity [69,70], which may lead to *DNMT1* downregulation. PTEN protein is a negative regulator of the intracellular oncogenic signaling pathways, including PI3K/AKT and MAPK/AP-1 [69,70]. The transcription factors E2F and AP-1 activate *DNMT1* expression due to the presence of binding sites in the *DNMT1* regulatory region [70,71,72]. Interestingly, Pabona et al. and Montales et al. showed that exposure to lunasin causes significant *PTEN* upregulation in breast and colon cancer cells [73,74] (Table 3).

Noteworthy, in RWPE-2, prostate epithelial cancer cells exposed to 2 µM lunasin for 24 h demonstrated an almost 6% increase in *p21* expression, with concomitant downregulation of *DNMT1* by 5%, compared to control cells. Unfortunately, as this study (accession number GSE2992) included only one sample per condition, it is impossible to verify the significance of those changes [19]. Further research is needed to assess lunasin as a potential regulator of DNA methylation processes (Figure 4).

**Table 3:** Lunasin and its impact on selected gene expression in breast cancer, colon cancer and leukemia cells.

Gene	Concentration	Exposure Time	Effects	Ref.
*BAX*	1 µM	24 h	2.8-fold increase in expression	[44]
	10 µM		6.6-fold increase in expression	
	50 µM		7.2-fold increase in expression	
	10 µM	24 h	2.2-fold increase in expression	[29]
*BCL-2*	1 µM	24 h	1.4-fold decrease in expression	[44]
	10 µM		2-fold decrease in expression	
	50 µM		2.8-fold decrease in expression	
	50 µM	24 h	2-fold decrease in expression	[75]
	10 µM	24 h	2-fold decrease in expression	[29]
*CASP3*	1 mg LES/mL (50 µM)	24 h	12-fold increase in expression	[30]
	10 µM	24 h	1.8-fold increase in expression	[29]
	40 and 80 µM	72 h	1.9-fold increase in expression	[76]
	1 µM	24 h	1.5-fold activity increase	[44]
	10 µM		1.6-fold activity increase	
	50 µM		1.8-fold activity increase	
*CASP8*	1 mg LES/mL (50 µM)	24 h	5-fold increase in expression	[30]
	10 µM	24 h	1.6-fold increase in expression	[49]
	75 µM			
*CASP9*	1 mg LES/mL (50 µM)	24 h	6-fold increase in expression	[30]
*p21*	1 µM	24 h	3.6-fold increase in expression	[44]
	10 µM		4.7-fold increase in expression	
	50 µM		7.3-fold increase in expression	
	10 µM	24 h	2.2-fold increase in expression	[29]
*p27*	10 µM	24 h	2.3-fold increase in expression	[29]
*nCLU*	1 µM	24 h	3.7-fold increase in expression	[44]
	10 µM		5.5-fold decrease in expression	
	50 µM		5.6-fold decrease in expression	
	50 µM	24 h	2-fold decrease in expression	[75]
*MALT1*	50 µM	24 h	1.9-fold decrease in expression	[75]
*PTEN*	2 µM	24 h	2.4-fold increase in expression	[73]
	2 µM	24 h	2.5-fold increase in expression	[74]

### 4.2. In Vitro and In Vivo Studies on Lunasin Chemopreventive Activity 

Lunasin, partly by its epigenetically mediated alterations in gene expression, has been shown to arrest the cell cycle, inhibit cell proliferation and induce apoptosis. In the vast majority of conducted studies, it was observed that lunasin inhibited the growth of cancer cells in a dose- and time-dependent manner (Table 4). 

Various studies obtained similar IC_50_ values (153–195 µM) for synthetic lunasin against MDA-MB-231 invasive breast cancer cells following 48 h incubation [41,42,49,52]. Higher concentrations of synthetic lunasin were needed to achieve 50% inhibition in MCF-7 cells following 48 h incubation, with the values differing significantly between the studies, viz., 232 µM [42] and almost 422 µM [41]. This variation may be due to differences in the cell culture conditions or the lunasin preparations. Interestingly, lunasin exposure did not affect the growth of MCF10A human breast epithelial cells, suggesting that lunasin selectively inhibits cancer cell growth without affecting normal cells [41,42]. The studies indicate that in breast cancer cells lunasin executes chemoprevention via inflammatory and estrogen-related molecule regulation, such as IL-6, COX-2, Ob-R, VEGF and ERα/β genes, and inhibited the aromatase gene and activity [41]. Lunasin suppresses the metastasis of breast cancer cells through integrin-mediated FAK/Akt/ERK and NF-κB signaling pathways followed by downregulation of the activity and expression of matrix metalloproteinase MMP-2/9 [42].

The chemopreventive effectiveness of lunasin in inhibiting cancer cell growth has been also observed in human and mouse melanoma cells. Exposure of A375 and B16-F10 cells to lunasin-enriched soy extract (LESE) resulted in dose-dependent anti-proliferative effects with estimated IC_50_ values being higher than 300 µM in both cases [77].

Dia et al. examined lunasin cytotoxicity in different colon cancer cells. It has been observed that lunasin (>90%) purified from defatted soybean flour caused dose-dependent cytotoxicity in KM12L4, HT-29, HCT-116 and RKO colon cancer cells and in their oxaliplatin-resistant (OxR) variants following 24 h incubation. Among these parental cell lines, lunasin most potently inhibited the growth of metastatic KM12L4 colon cancer cells, i.e., at the lowest IC_50_ (13.0 μM). A lunasin concentration as low as 1 μM caused almost 20% inhibition in KM12L4 metastatic colon cancer growth, while a concentration of 50 μM caused at least a 90% reduction in the viability of KM12L4 cells. However, <100 μM lunasin showed no cytotoxicity to normal human colon fibroblasts CCD-33Co [29]. In colon cancer cells, lunasin modified the expression of human extracellular matrix (ECM) and adhesion genes indicating the role of lunasin in angiogenesis and metastasis of cancer cells. Lunasin affected cell cycle progression, arresting the cell cycle at the G2/M phase accompanied by increased expressions of the cyclin-dependent kinase inhibitors p21 and p27. The increase in apoptotic cells was accompanied by the modification of expression of the Bcl-2 family of proteins with concomitant increased expression of the pro-apoptotic Bax, nCLU and caspase-3. Lunasin also downregulated integrins α_5_ and β_2_, MMP10a matrix metalloproteinase associated with metastasis and tumor growth acceleration [29].

The estimated IC_50_ values were much higher against HCT-116 cells despite longer exposure times, i.e., 107.5 ± 1.9 µM after 72 h of incubation [76] and 64.25 μM after 48 h, with the latter calculated based on a dose-response curve [78]. The higher IC_50_ value found in these studies may result from the use of synthetic or recombinant lunasin, respectively. Possible differences in the secondary and tertiary structures observed between the purified lunasin of natural origin and the synthetic peptide, as well as the presence of other ingredients in the natural preparations, could be responsible for the different levels of cell inhibitory potential.

The effect of highly purified soybean-derived lunasin on the proliferation of four different human non-small-cell lung cancer cell lines was examined by McConnell and colleagues. Exposure to lunasin resulted in a dose- and time-dependent inhibition in proliferation only in H661 cells. Exposure to lunasin of other non-small-cell lung cancer cell lines (H1299, H460 and A549) and normal bronchial epithelial cell lines (HBE135-E6E7 and BEAS-2B) resulted in little or no effect when incubated over 72 h even at the high concentration (100 μM) [43]. Although H661, H1299 and A549 cells exhibited a dose-dependent decrease in colony formation upon culture with lunasin, H460 cells showed no decrease in the total number of colonies formed upon lunasin incubation, but they did exhibit a dose-dependent decrease in colony size when exposed to lunasin, as H1299 did [43].

Moreover, purified lunasin and 27% *w/w* soybean extract of lunasin (LES) demonstrated dose-dependent anti-proliferative effects against L1210 murine leukemia cells. The estimated IC_50_ values for both preparations after 48 h of lunasin exposure were almost the same (14 and 16 μM, respectively) [30]. The authors also noticed that lunasin-enriched soy flour (LES) preparation had no cytotoxic effect in HL60 and HepG2 cells, even when using more than 30 times higher concentrations of lunasin equivalent [30]. These observations are consistent with those of previous studies in which no growth inhibition was noted against cell lines, such as Caco-2 [79], HepG2 [78,80], H1299, H460 and A549 [43]. This may indicate that the anti-proliferative effects of lunasin are dependent on cell type.

**Table 4:** The results of in vitro studies on the chemopreventive activity of lunasin in cancer cells.

Cell Line	Concentration	Time of Exposure	Type of Lunasin	Effects	Ref.
Human breast cancer cell line MCF-7	10 µM	72 h	Synthetic	No significant effect	[39]
Human breast cancer cell line MCF-7 MDA-MB-231	5–200 µM	24–72 h	Synthetic	Decrease in cell proliferation IC_50_ MCF-7–(48 h) 232 μM IC_50_ MDA-MD-231–(48 h) 153 μM	[42]
Human breast cancer cell line MDA-MB-231	10–200 µM	48 h	Synthetic	Decrease in cell proliferation IC_50_ MDA-MB-231–181 µM	[52]
Human breast cancer cell line MDA-MB-231	0.1–200 µM	72 h	Synthetic	Decrease in cell proliferation IC_50_ MDA-MB-231–181µM	[49]
Human breast cancer cell line MCF-7MDA-MB-231	1–320 µM	24, 48 h	Synthetic	Decrease in cell proliferation IC_50_ MCF-7–(24 h) 508.6 µM, (48 h) 431.9 µM IC_50_ MDA-MB-231–(24 h) 224.7 µM, (48 h) 194.9 µM	[41]
Human breast cancer cell line MDA-MB-231	1–100 µM	48 h	Recombinant	Decrease in cell proliferation IC_50_ MDA-MB-231–56.73 µM	[78]
Human colon cancer cell line HT-29	1–100 µM	24 h	Natural 90%	Decrease in cell proliferation IC_50_ HT-29–61.7 µM	[44]
Human colon cancer cell line: HT-29 KM12L4 RKO HCT-116	1–100 µM	24 h	Natural >90%	Decrease in cell proliferation IC_50_ HT-29–61.7 µM IC_50_ KM12L4–13 µM IC_50_ RKO–21.6 µM IC_50_ HCT-116–26.3 µM	[29]
Human colon cancer cell line HT-29 Caco-2	10–200 µM	24, 48, 72 h	Synthetic	Decrease in cell proliferation greatest for HT-29 cells after 72 h incubation at concentration of lunasin of 200 µM (23.8% of non-viable cells) No cytotoxicity effects in Caco-2 cells after 72 h exposure	[79]
Human colon cancer cell line HCT-116	2 µM	48 h	Synthetic	Decrease in cell proliferation Increase in the number of apoptotic cells	[74]
Human colon cancer cell line HCT-116	5–160 µM	72 h	Synthetic	Decrease in cell proliferation IC_50_ HCT-116–107.5 µM	[76]
Human colon cancer cell line Caco-2	0.5–25 µM	24 h	Synthetic	No significant effect	[81]
Human colon cancer cell line HCT-116 Human hepatoma cell line HepG2	1–100 µM	48 h	Recombinant	Decrease in cell proliferation IC_50_ HCT-116–64.25 µM No cytotoxicity for HepG2 cells	[78]
Human hepatoma cell line HepG2	0.5–50 µM	20 h	Synthetic	No significant effect	[80]
Mouse leukemia cell line L1210	1–80 µM	24 h	Natural 98%	Decrease in cell proliferation IC_50_–13.9 µM	[82]
Mouse leukemia cell line L1210	1–100 µM	24 h	Natural 98% 27% (LES)	Decrease in cell proliferation IC_50_–14 µM IC_50_–16 µM	[30]
Human non-small-cell lung cancer cell line: H661 H1299 H460 A549	1–100 µM	24, 48, 72 h	Natural 99%	Decrease in cell proliferation (H661 cell line) IC_50_ H661 cells–(72 h) 63.9 µM No cytotoxicity for other cell lines	[43]
Human gastric adenocarcinoma cell line AGS	10–200 µM	24, 48, 72 h	Synthetic	Decrease in cell proliferation (AGS cells) at concentrations of 100 µM–7% and 200 µM–15% (average percentage)	[79]
Mouse skin cancer cell line B16-F10 Human skin cancer cell line A-375	0.03–550 µM	24 h	Natural 40%	Decrease in cell proliferation IC_50_ B16-F10–330 µM IC_50_ A-375–370 µM	[77]

The in vivo studies presented in Table 5 using chemical carcinogens (DMBA, 7,12-dimethylbenz[*a*]anthracene; TPA, 12-*O*-tetradecanoylphorbol-13-acetate) or xenograft models have confirmed the chemopreventive effect of lunasin in breast, colon, lung, melanoma and skin cancer. Lunasin has been shown to prevent cell transformation in the presence of carcinogens, not only when it is administrated through intraperitoneal injection but also when applied topically in a mouse skin cancer model.

**Table 5:** The results of in vivo studies on the chemopreventive activity of lunasin.

In Vivo Model	Type of Cancer	Lunasin	Dose of Lunasin (#, Groups)	Administration Method	Scheme of Treatment	Effects	References
12-week-old female SENCAR mice	Mouse skin cancer initiated by DMBA (initiator) and TPA (promoter)	Synthetic	#1: control DMBA and TPA (n = 6); #2: solvents (n = 8); #3: 2.5 µg Lun/week (n = 9); #4: 25 µg Lun/week (n = 9); #5: 250 µg Lun/week (n = 9);	Percutaneous to the dorsal side, shaved	Before tumor induction 1 week 2 times a week After tumor induction 19 weeks 2 times a week	Reduction in skin tumor incidence by ~70%—dermal application of lunasin, 250 μg/week; decrease in tumor yield/mouse, and delay of the appearance of tumors by 2 weeks relative to the control 2.5 and 25 μg/week—decreasing trend in tumor incidence and yield (not statistically significant)	[23]
6-week-old athymic NCr-nu/nu mice	Human breast cancer (1 × 10^7^ MDA-MB-231 cells injected subcutaneously)	Natural	#1: control solvents; #2: Lun 20 mg/kg bw; #3: Lun 4 mg/kg bw (n = 8/group);	Intraperitoneal injection	Before cancer cells implantation 2 months 3 times a week	Decrease in tumor incidence by 49% at 20 mg/kg bw lunasin pretreated group and 33% at 4 mg/kg bw lunasin pretreated group compared with the vehicle-treated group	[83]
7- to 8-week-old male outbred homozygous nude mice	Human non-small-cell lung cancer (H1299 cells 2 × 10^6^ injected subcutaneously)	Natural	#1: control solvents; #2: Lun 30 mg/kg bw (n = 10/group);	Intraperitoneal injection	After cancer cells implantation 32 days 1 time a day	Reduction in tumor volume by 63% compared to the control group	[43]
6- to 8-week-old male athymic nude mice (Jackson #002019)	Human melanoma (A375 cells 2.5 × 10^6^ injected subcutaneously)	Natural	#1: control solvent (n = 8); #2: Lun 30 mg/kg bw (n = 10);	Intraperitoneal injection	After cancer cells implantation 34 days 1 time a day	Reduction in tumor volume by 55% and wet tumor weight by 46%	[55]
6-week-old male C57BL/6 mice	Mouse lung carcinoma (LLC cells 1 × 10^5^ injected subcutaneously)	Natural	#1: control solvents; #2: Lun 10 mg/kg bw; #3: Lun 30 mg/kg bw (n = 6–10/group);	Intraperitoneal injection	After cancer cells implantation 22 days 1 time a day	Inhibition of tumor growth initiated by LLC cells at 30 mg/kg dose of lunasin by 55%	[56]
	Mouse melanoma (B16-F0 cells 1 × 10^6^ injected subcutaneously)		#1: control solvents; #2: Lun 10 mg/kg bw; #3: Lun 30 mg/kg bw (n = 6–10/group);			Inhibition of tumor growth initiated by B16-F0 cells at the 30 mg/kg dose of lunasin by 60%	
6-week-old female Sprague Dawley (SD) rats	Breast cancer rat model induced by DMBA	Natural	#1: control solvent (n = 4); #2: control DMBA (n = 4); #3: tamoxifen 10 mg/kg bw (n = 4); #4: Lun 500 mg/kg bw (n = 4); #5: tamoxifen + Lun (10 + 500 mg/kg bw) (n = 4);	Intraperitoneal injection	After tumor induction 8 weeks	8 weeks of treatment shown that tamoxifen, lunasin and a combination of tamoxifen and lunasin could reduce tumor volume (>50%) and tumor weight when compared to control (DMBA)	[84]
6- to 8-week-old mice	Human colon cancer cells (spleen implantation of 1 × 10^6^ KM12L4 human colon cancer cells)	Natural	#1: 100 μL of PBS (n = 10); #2: Lun 4 mg/kg bw (n = 9);	Intraperitoneal injection By oral gavage	After cancer cells implantation 28 days 1 time a day	Reduction in CRC liver metastasis by 50% compared to the control group and the liver weight/body weight ratio by 23%	[85]
			#1: 100 μL PBS (n = 6); #2: Lun 8 mg/kg bw (n = 5); #3: Lun 20 mg/kg bw (n = 3);			Oral administration—reduction in the number of liver metastasis (not statistically significant)	

## 5. Conclusions

Studies indicate that lunasin inhibits histone acetylation, regulates the cell cycle, suppresses proliferation and induces apoptosis in cancer cells. Since lunasin is involved in the regulation of histone acetylation status and hereby affects the expression of multiple genes, it seems pivotal to perform more research on its potential role as a modulator of DNA methylation (Figure 2). Further research is necessary to identify the mechanism behind the epigenetic chemopreventive activity of lunasin. Even so, lunasin seems to be a promising bioactive anti-cancer agent and a potent epigenetic modulator.

## Figures and Tables

**Table 1:** Lunasin content in soybean and other plants.

Plant	Content of Lunasin (mg per g of Seeds)
Soybean	0.5–8.1
Barley	0.01–0.02
Wheat	0.2–0.3
Rye	0.045–0.15
